# Methods for transient assay of gene function in floral tissues

**DOI:** 10.1186/1746-4811-3-1

**Published:** 2007-01-08

**Authors:** Yongjin Shang, Kathy E Schwinn, Michael J Bennett, Donald A Hunter, Toni L Waugh, Nilangani N Pathirana, David A Brummell, Paula E Jameson, Kevin M Davies

**Affiliations:** 1 New Zealand Institute for Crop & Food Research Limited, Private Bag 11600, Palmerston North, New Zealand; 2 AgResearch, Private Bag 11008, Palmerston North, New Zealand; 3 Institute of Molecular BioSciences, Massey University, Private Bag 11222 Palmerston North, New Zealand; 4 School of Biological Sciences, University of Canterbury, Private Bag 4800, Christchurch, New Zealand

## Abstract

**Background:**

There is considerable interest in rapid assays or screening systems for assigning gene function. However, analysis of gene function in the flowers of some species is restricted due to the difficulty of producing stably transformed transgenic plants. As a result, experimental approaches based on transient gene expression assays are frequently used. Biolistics has long been used for transient over-expression of genes of interest, but has not been exploited for gene silencing studies. *Agrobacterium*-infiltration has also been used, but the focus primarily has been on the transient transformation of leaf tissue.

**Results:**

Two constructs, one expressing an inverted repeat of the *Antirrhinum majus *(Antirrhinum) chalcone synthase gene (*CHS*) and the other an inverted repeat of the Antirrhinum transcription factor gene *Rosea1*, were shown to effectively induce *CHS *and *Rosea1 *gene silencing, respectively, when introduced biolistically into petal tissue of Antirrhinum flowers developing *in vitro*. A high-throughput vector expressing the Antirrhinum *CHS *gene attached to an inverted repeat of the *nos *terminator was also shown to be effective. Silencing spread systemically to create large zones of petal tissue lacking pigmentation, with transmission of the silenced state spreading both laterally within the affected epidermal cell layer and into lower cell layers, including the epidermis of the other petal surface. Transient *Agrobacterium*-mediated transformation of petal tissue of tobacco and petunia flowers *in situ *or detached was also achieved, using expression of the reporter genes *GUS *and *GFP *to visualise transgene expression.

**Conclusion:**

We demonstrate the feasibility of using biolistics-based transient RNAi, and transient transformation of petal tissue via *Agrobacterium *infiltration to study gene function in petals. We have also produced a vector for high throughput gene silencing studies, incorporating the option of using T-A cloning to insert the gene sequence of interest. These techniques should allow analysis of gene function in a much broader range of flower species.

## Background

The proliferation of DNA sequences from EST and genome studies has driven an increasing interest in rapid assay systems as alternatives to stable transgenics for establishing gene function. Transient over-expression of gene sequences using biolistics (particle bombardment) is now well established for functional assays. In particular, it has been extensively applied in studies on plant pigmentation, using flower petals or developing maize seeds. However, this technique is limited in the range of tissues and biological systems to which it can be applied. Most notably, unless an obvious change in phenotype occurs, it is difficult to obtain a sufficient quantity of transformed cells to enable molecular or biochemical analysis of the impact of the transgene.

More recently, the use of *Agrobacterium tumefaciens *infiltration (agroinfiltration) for transient assays has become established for processes such as assigning gene function [e.g. [1-5]], promoter element analysis [6] and inducible gene studies [7]. The majority of results have been obtained using *Nicotiana benthamiana*, which is particularly suited to this method. However, the agroinfiltration transient assay system has recently been optimized for other species, including *Lactuca sativa *(lettuce), *L. serriola *(wild lettuce), *Solanum lycopersicum *(tomato) and some cultivars of *Arabidopsis thaliana *(Arabidopsis) [5]. Vegetative tissues have typically been used for agroinfiltration, although tomato fruit [8] and hairy root cultures (using *A. rhizogenes*) have also been used [9].

The development of RNA-interference (RNAi) gene silencing methods, based on the triggering of sequence-specific RNA degradation in a similar manner to antisense [10] or sense suppression [11,12] but with higher efficiency, has allowed the improvement of transient assay systems for loss of gene function [13-16]. Most of the RNAi systems have been with virus-induced gene silencing (VIGS), initially in *N. benthamiana *[17] and subsequently in other species as new viral vectors have been developed, for example for Arabidopsis [18], *Hordeum vulgare *(barley, [19]), *Pisum sativum *(pea, [20]), *Glycine max *(soyabean, [21]) and tomato [22]. However, for each target plant species a suitable virus vector must be identified, and even when a suitable viral species is known, its use may be limited by local biosecurity regulations.

Agroinfiltration has also been used as a delivery system for transient RNAi [3,9]. However, as with VIGS, agroinfiltration requires that the host is amenable to infection by the pathogen, and without the induction of tissue necrosis. Biolistic delivery offers an alternative delivery system that avoids the need for a pathogen and allows use of simple vectors lacking T-DNA or virus sequences. Other developments of agroinfiltration or RNAi technology include the suppression of gene silencing to allow higher levels of transgene expression [3], and the use of novel vector structures for higher throughput, such as the use of Gateway cloning [e.g. [23]] or vectors with an inverted repeat of the transcript termination sequence rather than the target gene sequence [24].

*Antirrhinum majus *(Antirrhinum) and petunia (most commonly *Petunia hybrida *and *Petunia *'Mitchell') are classic model systems, with growing EST and genomics resources. Both species have been used in studies of floral organ development, floral scent production, self-incompatibility and the biosynthesis and regulation of production of anthocyanin pigments in flowers (see reviews of Schwarz-Sommer et al. [25]; Gerats and Vandenbussche [26]). For Antirrhinum, although *Agrobacterium*-mediated systems are available [27], production of stable transgenics remains a difficult process. Thus, we were interested in establishing additional transient assay systems for these model species.

We report here the establishment of agroinfiltration for petunia and *N. tabacum *(tobacco) floral tissues, and the use of biolistics for transient RNAi in Antirrhinum. These systems have been applied to flowers *in situ *and flower buds cultured *in vitro*. In addition, we have developed and tested a new high-throughput vector for RNAi assays.

## Results and discussion

### Assay of gene function in flowers using transient RNAi

To enable the use of a sealed chamber biolistic apparatus, detached flower buds of Antirrhinum were used. We had previously determined that Antirrhinum buds could successfully develop *in vitro*, with buds 5–10 mm in length developing relatively normal pigmentation and expanding to open flowers, although the youngest buds did not always reach the normal size and developed more slowly.

**Figure 1:**
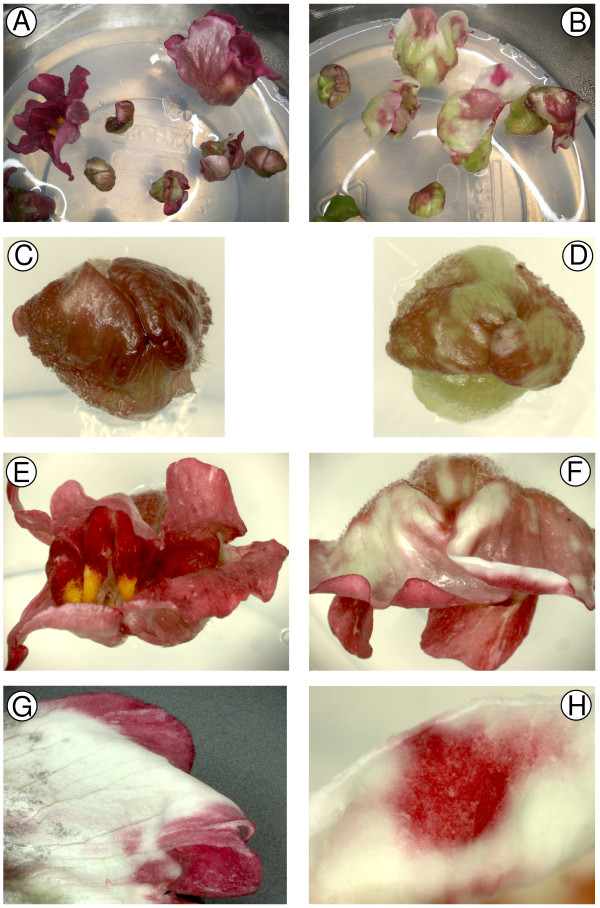
**Inhibition of the function of *CHS *in Antirrhinum flower buds using transient RNAi**. Flower buds (line 603) cultured *in vitro *are shown 3–14 days after the biolistic introduction of the control plasmid pRT99GFP (A, C and E) or the pRNA69-based plasmid pPN187 for *CHS *RNAi inhibition (B, D, F, G and H). C and D are 3 days post bombardment; A, B, E, F and H are 8 days post bombardment; G is 14 days post bombardment.

To test transient, biolistic-based RNAi for determining gene function in flowers, two flower colour genes were targeted. The construct pPN187, based on pRNA69, was made for formation of hairpin RNA of an Antirrhinum chalcone synthase transgene (*CHS*). *CHS *has been used as a test gene in many gene suppression studies, and was one of the first targets of sense and antisense RNA experiments in plants [11,12,28]. CHS is one of the biosynthetic enzymes of the anthocyanin pathway (catalyzing the first step committed to the biosynthesis of all flavonoids), and inhibition of this step in anthocyanin-accumulating flowers results in the easily detected phenotype of white petals. Flower buds from a fully pigmented Antirrhinum line were picked when 3–10 mm in length (stages 1 and 2 as defined by Martin *et al*. [29]), the sepals removed, and the outer epidermis of the exposed petals bombarded six times with pPN187. Buds at early developmental stages were used so that petal tissue was relatively un-pigmented when it was bombarded. As the buds subsequently developed in culture, notable differences in pigmentation were apparent in comparison to control buds, which were bombarded with pRT99GFP (Figure 1). While still at the unopened bud stage, extensive areas with markedly reduced pigmentation could be seen, with the chlorophyll of the young bud stage visible (Figure 1B and 1D). As the flowers developed, white patches appeared in the petals rather than the usual fully red colouration (Figure 1F, G and 1H), and these were seen in both the inner and outer epidermis, which are normally both pigmented in Antirrhinum. The areas lacking pigmentation did not have sharp boundaries, and mixed cell populations were apparent (Figure 1G and 1H).

**Figure 2:**
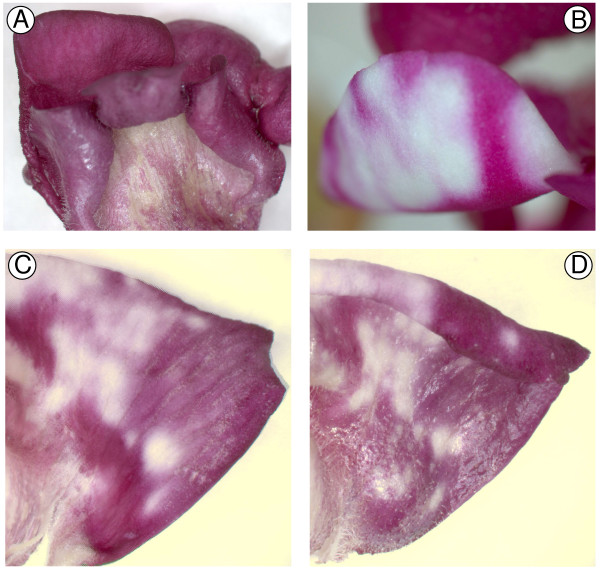
**Inhibition of *Rosea1 *activity in Antirrhinum flower buds using transient RNAi**. Petals of buds (line 522) cultured *in vitro *are shown 12–17 days after the biolistic introduction of the control plasmid pRT99GFP (A) or the pPN107 plasmid for *Rosea1 *RNAi inhibition (B, C and D). (C) and (D) show the inner and outer epidermis, respectively, of the same region of one petal. The same pattern of inhibition on both surfaces demonstrates that the silencing signal was transmitted from the bombarded outer epidermis to the inner epidermis.

The other colour gene targeted was *Rosea1*, which encodes a MYB-related transcription factor that regulates anthocyanin biosynthesis in Antirrhinum [30]. The construct used was pRNA69-based pPN107, for formation of hairpin RNA of an Antirrhinum *Rosea1 *transgene. As with the CHS experiments, biolistic introduction of the plasmid into Antirrhinum buds resulted in the development of white, non-pigmented areas of the petals (Figure 2B). These did not occur in buds bombarded with the control construct (Figure 2A). By comparing the pattern of pigment loss between the inner and outer epidermis, it was clear that, at least in many cases, where pigment was absent in one epidermis, it was also absent from the corresponding cells in the other epidermis (Figure 2C and 2D). While cell division in the bud continues until the bud is approximately 10 mm long [31], the zones of inhibition observed across both epidermal surfaces are not due to cell division from the original transformed cells, as bombardment was at a stage past the formation of independent epidermal cell lineages. Therefore, the presence of the same pattern of gene inhibition in cells of both the inner and outer epidermis suggests that the RNAi inhibition signal is moving out from the original biolistically transformed cells and moving between cell layers. Such a finding is consistent with the mobility of the silencing signal and spreading of the silencing state that has been observed in other species and tissues [32,33], and contrasts with the conclusions of Douchkov *et al*. [23], who assumed the method could be used to study only cell-autonomous traits. We do not know the extent to which the silencing signal was promulgated from an individual transformed cell, as the patterns of inhibition are most likely due to the merging of smaller zones of inhibition (due to the frequency of transformed cells that can be achieved in Antirrhinum petals using particle bombardment). The extent of inhibition observed means that it will be possible to use biolistics-based transient RNAi to produce significant amounts of transgenic tissue for subsequent analysis. With some genes of interest, it may be necessary or desirable to simultaneously inhibit expression of a marker gene to aid in the identification of areas of tissue for analysis. Chen *et al*. [34] used tandem constructs for CHS and an ACC oxidase gene in a viral vector to induce transient RNAi in a purple-flowered petunia. Silencing of CHS resulted in white flowers or flower sectors, and it was found that within these flowers or flower sectors transcript abundance from the target ACC oxidase gene (and a related ACC oxidase gene) was greatly reduced compared with abundance in purple tissue. Also, tandem inverted repeats within a vector to produce hairpin RNA for a selectable marker gene and a gene of interest were successful in triggering RNAi silencing of both genes in *Chlamydomonas*, thus allowing RNAi strains to be easily selected [35].

Although excised flower buds and a sealed chamber 'gene gun' were used in this study, it is assumed that the chamber-less guns would allow transient RNAi experiments with petals *in situ*.

### An improved vector for assay of gene function using RNAi

**Figure 3:**
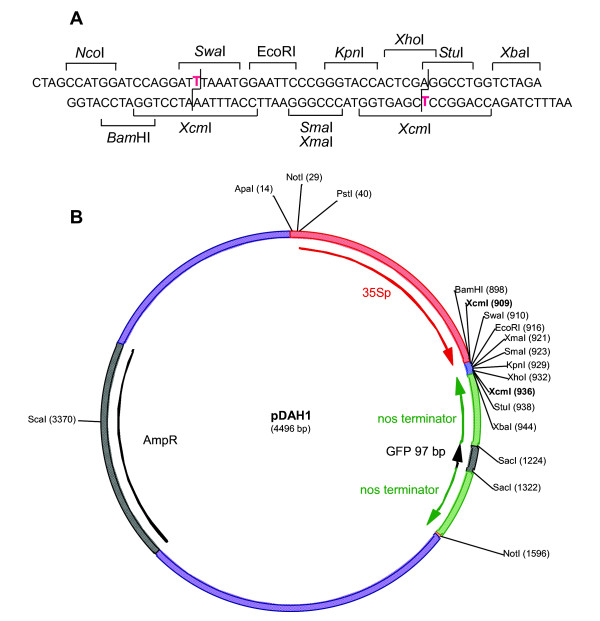
**Multiple cloning site (A) and plasmid map (B) of the high-throughput RNAi silencing vector pDAH1**. Abbreviations used are: 35Sp, CaMV 35S promoter; nos terminator, transcript termination region of the nopaline synthase gene of *A. tumefaciens*; GFP, green fluorescent protein; AmpR, gene for ampicillin resistance. NcoI site is not shown in (B) as it is not unique.

**Figure 4:**
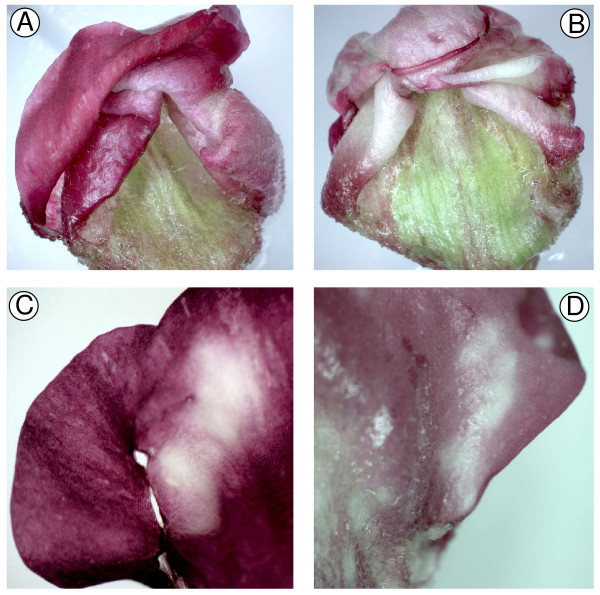
**Inhibition of the function of *CHS *in Antirrhinum flower buds using transient RNAi based on the plasmid vector pDAH1**. Petals of buds (line 522) cultured *in vitro *after the biolistic introduction of the control plasmid pRT99GFP (A) or the pDAH1-based plasmid pPN283 for *CHS *RNAi inhibition (B, C and D). (A) and (B) show buds 7 days after bombardment. (C) shows an example of inhibition in the inner epidermis, and (D) in the outer epidermis.

Construction of hairpin gene constructs can be a rate-limiting step for high-throughput screens of gene function, and more recently developed constructs have used a hairpin of the transcript terminator region for easier construct building [24]. To enable high-throughput construction of RNAi vectors, we made a new vector, pDAH1, that incorporates an antisense *nos*-sense *nos *hairpin and several other advantageous features (Figure 3). In particular; two XcmI restriction sites in the multiple cloning site (MCS) allow T-A cloning of the gene sequence of interest; NotI sites flank the promoter-hairpin sequence for cloning into pART27-based binary vectors [36]; the promoter can be exchanged using the upstream PstI site and any of the sites in the MCS; and the antisense *nos*-sense *nos *hairpin is separated by SacI sites to allow it to be replaced if required. The spacer in pDAH1 is 97 bp, which is close to the minimum size that can be used to separate hairpin-producing sequences. To test the utility of pDAH1, the ORF of Antirrhinum *CHS *was PCR-amplified using Taq polymerase and directly ligated into the MCS using T-A cloning, resulting in pPN283. Biolistic-based RNAi was then carried out using the same method as used for pPN187 and pPN107. The pPN283 construct was effective in inhibiting pigment formation in Antirrhinum buds (Figure 4), with zones of silencing observed as white patches, similar to those seen after bombardment with the construct containing an inverted repeat of the *CHS *transgene (Figure 1).

### Transient gene expression in petals using agroinfiltration

**Figure 5:**
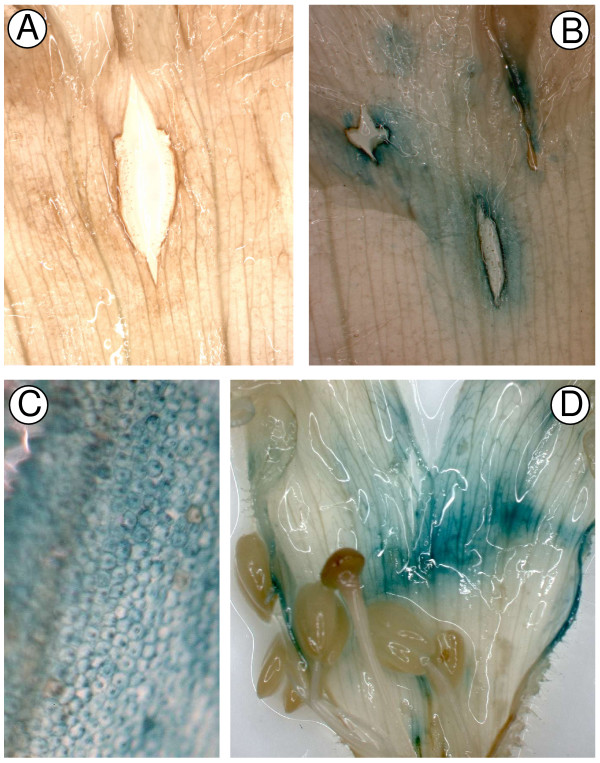
**Transient *GUS *gene expression in tobacco and petunia flowers using *Agrobacterium tumefaciens *infiltration**. Petals of tobacco (A, B and C) and petunia (D) were stained for GUS activity 1.5 to 3 days after infiltration with *A. tumefaciens *strain LBA4404 harbouring either the control plasmid pART27 (A) or the *35S:IGUS *construct p27IGUS (B, C and D). The petals were infiltrated while attached to the plant (A and B) or detached (C and D). Cuts were made in the petals to enable penetration of the GUS substrate.

**Figure 6:**
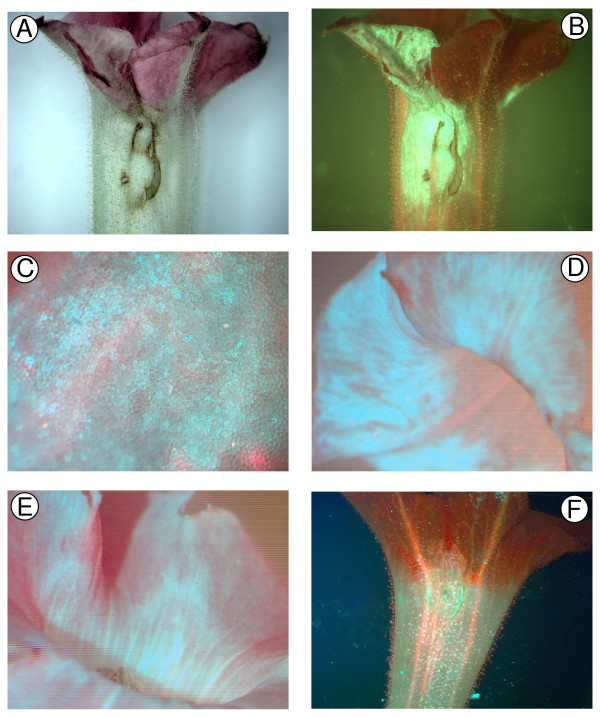
**Transient *GFP *gene expression in tobacco flowers using *Agrobacterium tumefaciens *infiltration**. Petals infiltrated with *A. tumefaciens *strain LBA4404 harbouring a *35S:GFP *construct when they were attached (A, B, C) or detached (D) are shown 2 to 2.5 days after infiltration. Panel E shows GFP fluorescence from a flower of a tobacco plant stably transformed with a *35S:GFP *construct. Panel F shows a flower from a control line transformed with an empty vector (pART27). Images are shown for petals under normal light (A) and blue light (B, C, D, E and F). GFP expression is seen as green fluorescence in B and green-blue fluorescence in C, D and E (the blue colour occurred with strong GFP fluorescence digitally photographed under higher magnification).

Agroinfiltration for transient gene expression was tested in three species, Antirrhinum, petunia and tobacco, using the intron-*GUS *(*IGUS*) and *GFP *reporter genes. The intron in the *GUS *gene prevents *Agrobacterium*-derived GUS expression. Tobacco flowers developed normally after agroinfiltration of flowers *in situ *with *35S:IGUS*, and GUS staining after 1.5 to 3 days clearly showed GUS expression throughout the epidermis of the petals (Figure 5). Infiltration was successful also for detached tobacco flowers (Figure 5C), and similar results were observed when using GFP as the reporter (Figure 6). Infiltrating detached petunia flowers with *35S:IGUS *(Figure 5D) and *35S:GFP *(data not shown) constructs showed similar results as for tobacco, with strong positive signal for samples ranging from 15 mm-length buds to fully opened flowers. The level of GFP detected in tobacco petals infiltrated with a *35S:GFP *construct was visually similar to that of transgenic plants stably transformed with a *35S:GFP *transgene (Figure 6D compared with 6E). Examinations based on petal sections revealed that the reporter genes were expressed in all cell layers of the agroinfiltrated petals (data not shown). GUS or GFP signal was not observed in any of the respective negative control plants (Figures 5A and 6F; data not shown).

Agroinfiltration (using strain LBA4404) of Antirrhinum petals was unsuccessful (data not shown). Both lack of *Agrobacterium *infection and induced necrosis in target tissues have been noted as problems when developing agroinfiltration protocols, and the identification of *Agrobacterium *strains more compatible with the host plant has been successful for some species [5]. There are no previous reports on the use of floral tissue for transient assays with agroinfiltration, so it is not known whether lack of success with transient infiltration can be correlated to the infectability of species with *Agrobacterium *when generating stably transformed plants. Antirrhinum is readily infected by *A. tumefaciens *or *A. rhizogenes *[27]; however, regeneration of plantlets from transformed tissues is difficult.

## Conclusion

Biolistics-based transient RNAi in floral tissues was demonstrated for the classic model species Antirrhinum, and agroinfiltration methods for transient gene expression were successfully established for floral tissues of two other model species, tobacco (*N. tabacum*) and petunia. To our knowledge, this is the first report describing the application of these techniques to floral tissue. These methods should allow analysis of gene function in a broader range of flower species. Furthermore, a construct was developed for high-throughput RNAi silencing and successfully tested in petals of Antirrhinum.

## Methods

### Plant material

The 'Mitchell' petunia line (sometimes referred to as W115) was obtained from the University of Auckland, New Zealand, and is *Petunia axillaris *× (*P. axillaris *× *P. hybrida*) [37]. Antirrhinum line 603 and wild type lines H75A and JI522 were obtained as seed from Prof Cathie Martin and Rosemary Carpenter of the John Innes Centre, Norwich (UK). The tobacco line used was *N. tabacum *cv Samsun.

### pDAH1 vector construction

The plasmid DAH1 (Figure 3) was derived from pGEM5Zf (In Vitro Technologies, Auckland, New Zealand). Linker1F and Linker1R (5'-NotI-PstI-MfeI-NotI-3') were inserted into the SphI/NsiI site of pGEM5Zf, destroying the SphI and NsiI sites, to produce pAF. A PstI-EcoRI fragment containing the CaMV 35S promoter from pART7 [36], Linker2F and Linker2R (BamHI/SacI) and the *nos *terminator were then ligated into the PstI/MfeI sites of pAF to produce pAO. A second *nos *terminator fragment (with 97 bp of the 3'-end of the GFP sequence) was PCR-amplified with the primers BglII-SGFP-NOS and XbaI-EcoRI-ASNOS, digested with BglII and XbaI and cloned in the antisense orientation into the XbaI/BamHI sites of pAO to make the *nos *hairpin vector pAP. A multiple cloning site was then created by inserting Linker3F (Figure 3A, top strand) and Linker3R (Figure 3A, lower strand) into pAP digested with XbaI and EcoRI (destroying the upstream XbaI site) to produce pDAH1. The oligonucleotide sequences used were:

Linker1F 5'-AGCGGCCGCCTGCAGACGGACAATTGGCGGCCGCCTGCA-3'

Linker1R 5'-GGCGGCCGCCAATTGTCCGTCTGCAGGCGGCCGCTCATG-3'

Linker2F 5'-CCTGAG-3'

Linker2R 5'-GATCCTCAGGAGCT-3'

Primer BglII-SGFP-NOS 5'-CGCAGATCTCCACATGGTCCTTCTTGA-3'

Primer XbaI-EcoRI-ASNOS

5'-TGCTCTAGAACGAATTCCCGATCTAGTAACATA-3'

### *Agrobacterium*-infiltration vectors

The binary vectors used were pBINm-gfp5-ER [38,39] and p27IGUS, a vector based on pART27 but containing a *GUS *reporter gene with an intron (*IGUS*; [40]). *IGUS *was PCR-amplified from pMOG410 and cloned into pART7 which had been digested with KpnI and SmaI. The cassette containing *35S:IGUS:OCS *was released by NotI digestion and ligated into NotI-digested pART27.

### Hairpin vectors for dsRNA

cDNA encoding the ORF of *CHS *was PCR-amplified from a pool of first strand cDNA derived from floral RNA (isolated from Antirrhinum wild type line H75A) using FastStart Taq polymerase (Roche Applied Science) and gene-specific primers. pPN187 was constructed based on the vector pRNA69 [41], containing the CaMV 35S promoter, multiple cloning sites (separated by the *Yabby5 *intron) for inserting sense and antisense sequences for the gene of interest, and the *ocs *terminator. The *CHS *ORF was ligated in a sense orientation into the XhoI site and in an antisense orientation using ClaI/XbaI sites. pPN283 was constructed by ligating the PCR-amplified *CHS *ORF into pDAH1 using T-A cloning. pPN107 was made by ligating the ORF of *Rosea1 *into pRNA69 in a sense orientation using the XhoI/BclI sites and in an antisense orientation using the ClaI/XbaI sites.

### *In vitro *culture of Antirrhinum floral buds

Whole buds (3–10 mm in length; minus sepals) were surface sterilized for 10 minutes using 10% (v/v) bleach containing 1–2 drops of Tween20/100 mL. Buds were then rinsed three times with sterile water and maintained on medium #2 (1/2 × MS macro salts/L; 1 × MS micro salt/L; 1 × MS iron/L; 1 × LS vitamins/L; 3% sucrose (w/v)/7.5% agar (w/v) during and after particle bombardment. The cultured buds were placed under artificial lights (16 h photoperiod) at 25°C after bombardment.

### Particle bombardment of floral buds

Particle bombardment used a helium-driven particle inflow gun based on Vain *et al*. [42], but modified by the addition of a high speed, direct current solenoid valve for accurate valve opening times down to 8 ms. The bombardment conditions were a solenoid valve opening time of 30 ms, a pressure setting of 400 kPa, a shooting distance of 13 cm, and a partial vacuum of approximately -95 kPa. Preparation of the DNA/gold suspension was essentially as in Schwinn *et al*. [30], with the gold in 50 μL water prior to precipitation of plasmid DNA onto the gold particles. A final DNA concentration (for each construct of interest) of 2 μg DNA per mg of 1.0 μm gold particles was used. Each bombardment used 5 μL of DNA/gold suspension. Buds were bombarded six times and then cultured. Transformation was monitored by including an internal control vector, pRT99GFP, which was co-precipitated onto the gold particles with the construct of interest (at one-fifth the concentration). Also, pRT99GFP alone (at the same concentration) was used for control bombardment experiments.

### *Agrobacterium *infiltration of floral tissue

Attached tobacco flowers were infiltrated with *A. tumefaciens *strain LBA4404 harbouring either p27IGUS or pBINm-gfp5-ER. The LBA4404 cells were cultured in 10 ml LB broth with antibiotic overnight, pelleted and re-suspended in medium #1003 (AB media salts + NaH_2_PO_4 _240 mg/L+ glucose 10 g/L + MES 14.693 g/L) supplemented with 100 μM acetosyringone, and cultured for 4 h. The cells were then pelleted and re-suspended to a concentration of A_600 _= 0.5 in 1% (w/v) glucose solution (pH 5.3) supplemented with 100 μM acetosyringon. Flower buds or opened flowers were pierced with a needle and infiltrated with the *A. tumefaciens *culture using a syringe. When using detached flowers, the flowers were cut into half across the middle of the tube, agroinfiltrated using a vacuum chamber, blotted with Whatman paper and cultured in petri-dishes containing moistened Whatman paper. The agroinfiltrated, detached flowers were cultured at 25°C under artificial lights (16 h photoperiod) for 2 to 2.5 days before examining reporter gene activity.

### Reporter gene assays

1.5 to 3 days following agroinfiltration, flowers were histochemically assayed for GUS activity. Flower samples were incubated for 12 to 48 h at 37°C in X-gluc staining buffer (5-bromo-4-chloro-3-indoyl-β-D glucuronide dissolved in dimethyl formamide then diluted to 0.5 mg/L X-gluc in 50 mM phosphate buffer (pH 7.0) containing 1% (v/v) Triton X-100), and then placed in 70% (v/v) ethanol to remove the chlorophyll and preserve the sample. In some instances a brown colour developed due to tissue necrosis, and this was removed by treatment in a 5% acetic acid/ethanol (v/v) solution at 70°C for 30 min. To enable penetration of the GUS substrate into the petals they were either cut into pieces or wounds were made in the petals.

Light microscopy used an Olympus BH2 microscope and fluorescent microscopy used an Olympus SZX microscope. Images were recorded using a Leica DC 50 digital camera.

## Competing interests

The author(s) declare that they have no competing interests.

## Authors' contributions

YS conceived of the agroinfiltration project, made the constructs and conducted the experiments. KES conceived of and managed the RNAi project, contributed to the experiments, and co-wrote the manuscript. MJB made pPN107 and pPN187, conducted biolistic experiments with these constructs, and modified the bombardment conditions. DAH suggested using TA-cloning as a way of making the inverted nos construct a high throughput vector, and designed and made pDAH1. TLW made pPN283 and conducted biolistic experiments with this construct. NNP conducted biolistic experiments with pPN107 and pPN283. DAB designed the polylinker of Fig 3A, contributed to the design of pDAH1, and contributed to and edited the manuscript. PEJ provided overall academic guidance to YS during his PhD study. KMD drafted the manuscript and provided project coordination.
